# Active Sensing and Its Application to Sensor Node Reconfiguration

**DOI:** 10.3390/s141018484

**Published:** 2014-10-08

**Authors:** Sooyong Lee

**Affiliations:** Department of Mechanical and System Design Engineering, Hongik University, Seoul 121-791, Korea; E-Mail: sooyong@hongik.ac.kr; Tel.: +82-2-320-1609; Fax: +82-2-322-7003

**Keywords:** perturbation/correlation, active sensing, gradient estimation, sensor node configuration

## Abstract

This paper presents a perturbation/correlation-based active sensing method and its application to sensor node configuration for environment monitoring. Sensor networks are widely used as data measurement tools, especially in dangerous environments. For large scale environment monitoring, a large number of nodes is required. For optimal measurements, the placement of nodes is very important. Nonlinear spring force-based configuration is introduced. Perturbation/correlation-based estimation of the gradient is developed and it is much more robust because it does not require any differentiation. An algorithm for tuning the stiffness using the estimated gradient for node reconfiguration is presented. The performance of the proposed algorithm is discussed with simulation results.

## 1. Introduction

The recent Fukushima nuclear disaster stressed that it is getting more and more important to explore and monitor areas that cannot be accessed by humans. One of the solutions is to install a sensor network and use the network to collect information. For instance, a sensor network is very useful for monitoring volcano activity by measuring temperature, pressure, sound and vibration [1]. Wireless sensor networks are being used widely, not only for military surveillance, but also for process monitoring, machine diagnosis, building automation and traffic control [2–4]. Each sensor node is relatively cheap and a large number of nodes are laid over a wide area. Therefore, efficient placement and dispatch of sensors are critical issues because the cost and detection capability are affected.

Previous work focused on sensor coverage and connectivity while trying to minimize the number of required sensors. In [5], the deployment of wireless sensor network in a deterministic model with obstacles is investigated. The sensor’s sensing model and coverage quality evaluation are developed for optimal sensor placement. Reference [6] proposed a self-organized algorithm, by waking up some sensor nodes or making some sensor nodes sleep. This algorithm used the virtual potential field to make the sensor nodes change positions. Another approach is to randomly deploy sensors based on the Poisson distribution in [7] using the relationship between the coverage area and node density. General optimization algorithms such as the simulated annealing algorithm [8] and the particle swarm optimization [9] are used for sensor placement. This paper presents a new idea of sensor node reconfiguration based on the sensor measurements, especially their spatial gradient.

**Figure 1.**
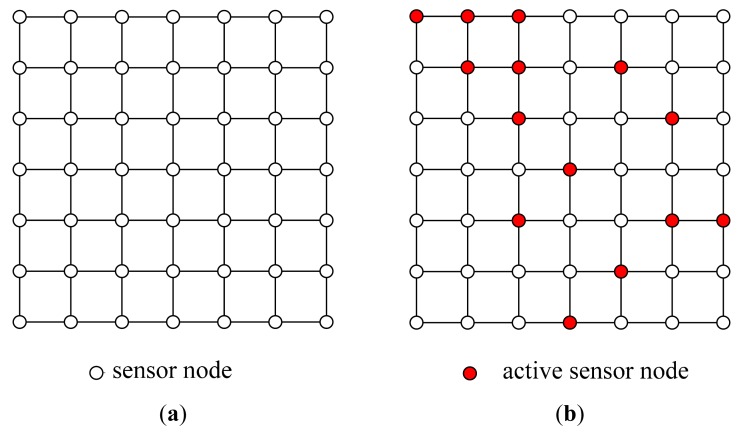

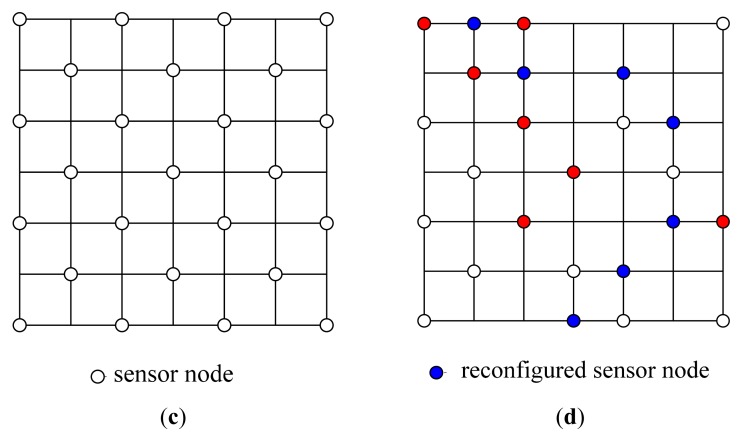
Sensor network. (**a**) Sensor network; (**b**) Active sensor nodes; (**c**) Sensor network; (**d**) Reconfigured nodes.

As the area to be monitored gets larger, the number of sensor nodes grows. Thereby the power source and the range of the wireless communication become more important. In case humans can’t reach an area to install the sensor network, it is also used to deploy the sensor nodes from the air. A more efficient way of using a sensor network is enabling only the necessary nodes in order to save power and reduce the communication load. For example, a sensor network with 7 × 7 nodes (Figure 1a) is composed of 49 nodes and only some of the nodes (Figure 1b) are activated to collect information. The selection of nodes is carefully planned to get the maximum throughput while minimizing the power consumption with given constraints. Another way of managing the sensor network is the reconfiguration of the nodes using the nodes with mobility. Figure 1c shows a sensor network with a less number of nodes than the one in Figure 1a. For the same set of nodes to be used as in Figure 1b, some nodes (red) are selected, and some nodes (blue) are moved. The goals of reconfiguration are maximizing the sensitivity and the resolution, while minimizing the operating power consumption and the total time of measurements.

We propose to use the change in measurement, with respect to the node movement; that is, the spatial gradient of the measurement, for reconfiguration of the nodes. For areas with relatively small (or almost zero) magnitude of the gradient, the changes in the measurement are negligible and the nodes are deployed sparsely. On the other hand, the nodes are placed densely where the magnitude of the gradient is large.

**Figure 2.**
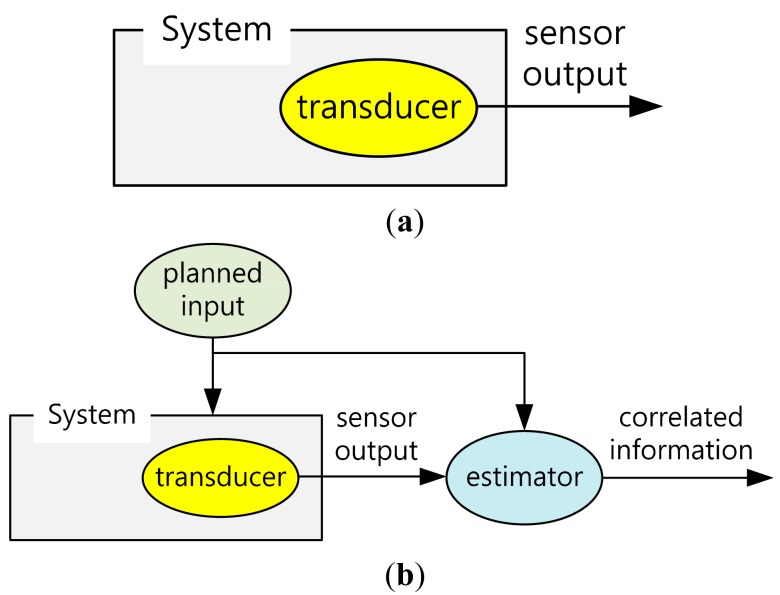
Active sensing. (**a**) Conventional sensing; (**b**) Estimation of correlation of sensor outputs with the planned inputs.

The key idea of getting the gradient of the measurement is, so called “active sensing”. Conventional sensing is composed of putting in a transducer where we want to measure the interested property and then getting the transducer outputs as in Figure 2a. Different from conventional sensing, the planned inputs are given to the system and the correlations between the given inputs and the transducer outputs are investigated. Instead of passively getting the transducer outputs, we first apply inputs to the system while measuring the responses, and then extract more copious information from the correlation.

In the following section, the correlations between the planned input and the transducer outputs are analyzed. With sinusoidal perturbation as the planned input, it is proved that the correlation value is related to the gradient of the output with respect to the input. Statistical analysis is described in Section 3, and the sensor node configuration is in Section 4, with the simulation results.

## 2. Active Sensing

In order to estimate the gradient of a function, we propose the perturbation/correlation based approach. In [10], a similar approach is used for multiple robot formation control. We first derive the fundamental relation and then verify the proposed method with several types of functions. Firstly, for a function, *F* of an input, *x*:

(1) F=F(x)

we add the perturbation, *δ*(*t*) to the nominal input value, *x*_0_ as:

(2) $$x\left(t\right)=x_{0}+\delta \left(t\right)$$

Usually a continuous and differential function is used for the perturbation, such as a sinusoidal function. For example, a sin function is used as in Equation (3) with an amplitude, *ε* and a frequency, *ω*:

(3) δ(t)=ɛsin(ωt)

The correlation value: *R* is defined as follows:

(4) $$R=\int_{t}^{t+\overset{2\pi }{\;}/\underset{\omega }{\;}}\delta \left(\tau \right)F\left(x\left(\tau \right)\right)d\tau$$

Similarly, in discrete time system, the correlation value, *Ȓ* is defined as:

(5) $$\overset{⌢}{R}=\sum_{i=1}^{2N}ɛ\sin\left(\frac{\pi }{N}i\right)F\left(x(t_{i})\right)$$

The representation of the input variable, *x* in discrete time is:

(6) $$x(t_{i})=x_{o}+ɛ\sin\left(\frac{\pi }{N}i\right);i=1,\ldots ,2N$$

Therefore, the discrete time correlation value is represented as:

(7) $$\overset{⌢}{R}=\sum_{i=1}^{2N}ɛ\sin\left(\frac{\pi }{N}i\right)F\left(x_{o}+ɛ\sin\left(\frac{\pi }{N}i\right)\right)$$

We apply the linear regression to derive the relation between the correlation value and the slope of a linear function, which is the gradient. From the linear regression analysis with the data set of (*x*(*t_i_*), *F*(*x*(*t_i_*)); *i* = 1, …, 2*N*), the slope of the function, *K* is derived as:

(8) $$K=\frac{\sum_{i=1}^{2N}\left(\left[x\left(t_{i}\right)-\bar{x}\right]\left[F\left(x\left(t_{i}\right)\right)-\bar{F}\right]\right)}{\sum_{i=1}^{2N}{\left(x\left(t_{i}\right)-\bar{x}\right)}^{2}}$$

where $\bar{x}=\frac{1}{2N}\overset{2N}{\underset{i=1}{\text{∑}}}x\left(t_{i}\right)$ and $\bar{F}=\frac{1}{2N}\overset{2N}{\underset{i=1}{\text{∑}}}F\left(x(t_{i})\right)$.

With Equations (7) and (8) becomes:

(9) $$K=\frac{\sum_{i=1}^{2N}ɛ\sin\left(\frac{\pi }{N}i\right)\left[F\left(x\left(t_{i}\right)\right)-\bar{F}\right]}{\sum_{i=1}^{2N}{ɛ}^{2}\sin^{2}\left(\frac{\pi }{N}i\right)}$$

and the second term in the numerator and the denominator become:

(10) $$\sum_{i=1}^{2N}\bar{F}ɛ\sin\left(\frac{\pi }{N}i\right)=0$$

(11) $$\sum_{i=1}^{2N}{ɛ}^{2}\sin^{2}\left(\frac{\pi }{N}i\right)=N{ɛ}^{2}$$

respectively. Hence the slope of the function from the linear regression analysis is represented as:

(12) $$K=\frac{\sum_{i=1}^{2N}F\left(x(t_{i})\right)\sin\left(\frac{\pi }{N}i\right)}{Nɛ}$$

And by comparing it with Equation (7), we derive the relation in-between as:

(13) $$\overset{⌢}{R}=n{ɛ}^{2}K$$

Hence, alternatively:

(14) $$K=\frac{\overset{⌢}{R}}{n{ɛ}^{2}}$$

Equation (14) is the one used to estimate the slope (gradient) of a function from the value of correlation.

Secondly, we apply the proposed perturbation/correlation to a function with two input variables, *x, y*:

(15) F=F(x,y)

From Taylor series expansion, the function is represented as:

(16) $$F\left[x\left(\tau +\Delta t\right),y\left(\tau +\Delta t\right)\right]=F\left[x\left(\tau \right),y\left(\tau \right)\right]+\delta_{x}\left(t\right){\frac{\partial F}{\partial x}|}_{\tau }+\delta_{y}\left(t\right){\frac{\partial F}{\partial y}|}_{\tau }$$

Two orthogonal functions are applied to the input variables concurrently:

(17) $$\delta_{x}={ɛ}_{x}\sin(\omega t)$$

(18) $$\delta_{y}={ɛ}_{y}\cos(\omega t)$$

where *ε_x_, ε_y_* are the amplitudes of each perturbation function respectively and *ω* is the common frequency.

The correlation between the input variable *x* and the function, *F* is represented as:

(19) $$\bar{\delta_{x}F}=\int_{\tau -\frac{2\pi }{\omega }}^{\tau }\delta_{x}F\left[x(\tau ),y(\tau )\right]dt+\int_{\tau -\frac{2\pi }{\omega }}^{\tau }{\delta_{x}}^{2}{\frac{\partial F}{\partial x}|}_{\tau }dt+\int_{\tau -\frac{2\pi }{\omega }}^{\tau }\delta_{x}\delta_{y}{\frac{\partial F}{\partial y}|}_{\tau }dt$$

As in the previous case of the first input function, some of the terms in Equation (18) become simplified as:

(20) $$\int_{\tau -\frac{2\pi }{\omega }}^{\tau }\delta_{x}dt=0$$

(21) $$\int_{\tau -\frac{2\pi }{\omega }}^{\tau }{\delta_{x}}^{2}dt=\frac{\pi {{ɛ}_{x}}^{2}}{\omega }$$

and due to the orthogonality of sin and cos functions:

(22) $$\int_{\tau -\frac{2\pi }{\omega }}^{\tau }\delta_{x}\delta_{y}dt=0$$

Therefore, Equation (18) becomes:

(23) $$\bar{\delta_{x}F}=\frac{\pi {{ɛ}_{x}}^{2}}{\omega }{\frac{\partial F}{\partial x}|}_{\tau }$$

and the gradient of the function *F* with respect to the input variable *x* is:

(24) $${\frac{\partial F}{\partial x}|}_{\tau }=\frac{\omega }{\pi {ɛ}_{x}^{2}}\bar{\delta_{x}F}$$

Similarly, the gradient of the function *F* with respect to the input variable *y* is represented as:

(25) $${\frac{\partial F}{\partial y}|}_{\tau }=\frac{\omega }{\pi {ɛ}_{y}^{2}}\bar{\delta_{y}F}$$

For functions with more than two inputs, the perturbations are selected with consideration of the orthogonality. In case a function with three inputs and the perturbations are given to two input variables of a function, the effects of the perturbed inputs to the remaining input (not perturbed), is considered. Let *ξ* be the unperturbed input, then the function is expanded as:

(26) $$\begin{array}{l} F\left[x\left(\tau +\Delta t\right),y\left(\tau +\Delta t\right),\xi \left(\tau +\Delta t\right)\right]=\\ F\left[x\left(\tau \right),y\left(\tau \right),\xi \left(\tau \right)\right]+\delta_{x}\left(t\right){\frac{\partial F}{\partial x}|}_{\tau }+\delta_{y}\left(t\right){\frac{\partial F}{\partial y}|}_{\tau }+\delta_{\xi }\left(t\right){\frac{\partial F}{\partial \xi }|}_{\tau }+\vartheta (2) \end{array}$$

The correlations are approximated as:

(27) $$\begin{array}{ll} \bar{\delta_{x}F}\approx & \int_{\tau -\frac{2\pi }{\omega }}^{\tau }F\left[x(\tau ),y(\tau ),\xi (\tau )\right]\delta_{x}(t)dt\\ & +\int_{\tau -\frac{2\pi }{\omega }}^{\tau }{\delta_{x}}^{2}{\frac{\partial F}{\partial x}|}_{\tau }dt+\int_{\tau -\frac{2\pi }{\omega }}^{\tau }\delta_{x}\delta_{y}{\frac{\partial F}{\partial y}|}_{\tau }dt+\int_{\tau -\frac{2\pi }{\omega }}^{\tau }\delta_{x}\delta_{\xi }{\frac{\partial F}{\partial \xi }|}_{\tau }dt \end{array}$$

Two input variables, *x, y* are perturbed as in Equations (17) and (18) using orthogonal functions. Then, Equation (26) becomes:

(28) $$\bar{\delta_{x}F}\approx 0+\frac{{ɛ}_{x}^{2}\pi }{\omega }{\frac{\partial F}{\partial x}|}_{\tau }+0+{\frac{\partial F}{\partial \xi }|}_{\tau }\int_{\tau -\frac{2\pi }{\omega }}^{\tau }\delta_{x}\delta_{\xi }dt$$

Therefore, if the following condition is satisfied, then Equation (23) is valid:

(29) $$\left|{\frac{\partial F}{\partial \xi }|}_{\tau }\int_{\tau -\frac{2\pi }{\omega }}^{\tau }\delta_{x}\delta_{\xi }dt\right|\ll \left|\frac{{ɛ}_{x}^{2}\pi }{\omega }{\frac{\partial F}{\partial x}|}_{\tau }\right|$$

## 3. Sensor Noise and Uncertainty

The noise in sensing is inevitable and it usually causes trouble. Also the uncertainty in sensing information should be considered. In [11], this issue is investigated for internal combustion engine tuning. We use the student *t*-distribution for this purpose. The student *t*-distribution is useful when the population variance is not known and especially the sample size is small.

For a normal set of *X*_1_, *X*_2_,…, *X_n_* ∼ *N* (*μ, σ*^2^), where *X̄_n_* is the sample mean, and sample variance is $S_{n}^{2}$. For a degrees of freedom *n* − 1, the *t*-distribution is defined as:

(30) $$t=\left({\bar{X}}_{n}-\mu \right)\frac{\sqrt{n}}{S_{n}}$$

For instance, 90% confidence level range is:

(31) $${\bar{X}}_{n}\pm t_{\left(0.05,n-1\right)}\frac{S_{n}}{\sqrt{n}}$$

By including the noise, *X*, the function with an input is described from the Taylor series expansion:

(32) $$F\left[x\left(\tau +\Delta t\right)\right]+X(\tau +\Delta t)=F\left[x\left(\tau \right)\right]+\delta_{x}\left(t\right){\frac{\partial F}{\partial x}|}_{\tau }+\delta_{y}\left(t\right){\frac{\partial F}{\partial y}|}_{\tau }+X(\tau )$$

A sin function is used for perturbation and the noise is represented with a variable, Δ*X* as:

(33) ΔX=X(τ+Δt)-X(τ)

The sample mean with 90% confidence level is represented as:

(34) $$-t_{\left(0.05,n-1\right)}\frac{S_{n}}{\sqrt{n}}\leq {\bar{X}}_{n}\leq +t_{\left(0.05,n-1\right)}\frac{S_{n}}{\sqrt{n}}$$

and then, the maximum value of Δ*X* is:

(35) $$\left|\Delta X\right|=2t_{\left(0.05,n-1\right)}\frac{S_{n}}{\sqrt{n}}$$

With considering the noise, the correlation between the input variable, *x* and the function, *F* is

(36) $$\bar{\delta_{x}F}=\int_{\tau -\frac{2\pi }{\omega }}^{\tau }\delta_{x}F\left[x(\tau )\right]dt+\int_{\tau -\frac{2\pi }{\omega }}^{\tau }{\delta_{x}}^{2}{\frac{\partial F}{\partial x}|}_{\tau }dt+\int_{\tau -\frac{2\pi }{\omega }}^{\tau }\delta_{x}2t_{\left(0.05,n-1\right)}\frac{S_{n}}{\sqrt{n}}dt$$

The last term in the above equation has its maximum value when the sign of the noise is same as the perturbation function, sin for a period. Hence, the maximum value is:

(37) $$\left|\int_{\tau -\frac{2\pi }{\omega }}^{\tau }\delta_{x}2t_{\left(0.05,n-1\right)}\frac{S_{n}}{\sqrt{n}}dt\right|=8{ɛ}_{x}t_{\left(0.05,n-1\right)}\frac{S_{n}}{\sqrt{n}}$$

Therefore, the gradient of *F* with respect to the input variable, *x* with the presence of the noise is:

(38) $${\frac{\partial F}{\partial x}|}_{\tau }=\frac{\omega }{\pi {ɛ}_{x}^{2}}\bar{\delta_{x}F}\pm \frac{8\omega }{\pi {ɛ}_{x}}t_{\left(0.05,n-1\right)}\frac{S_{n}}{\sqrt{n}}$$

We can derive the condition for neglecting the effects of sensor noise as:

(39) $$\left|8t_{\left(0.05,n-1\right)}\frac{S_{n}}{\sqrt{n}}\right|\ll \left|\frac{1}{{ɛ}_{x}}\bar{\delta_{x}F}\right|$$

*i.e.*, the effects becomes small when the standard deviation of the noise is small, or the degrees of the freedom is high, or the amplitude of the perturbation is small.

## 4. Configuration of Sensor Nodes

The configuration of sensor nodes is how to locate each sensor node appropriately. It is necessary to put the nodes densely in the area where the change of the interested physical property is large in order to increase the sensing resolution. With a limited number of nodes, it is very important to efficiently allocate the nodes to get the maximum sensing throughput. It is also necessary to constantly update the location of each node as the environment changes.

**Figure 3.**
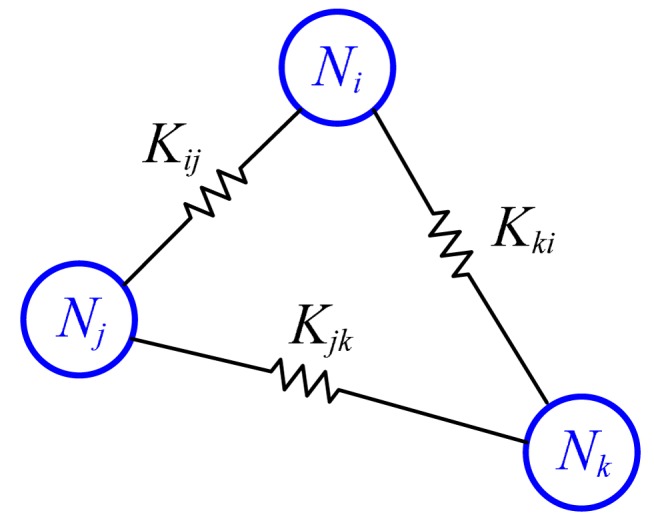
Interconnected nodes with springs.

This is similar to path planning of multiple robots for exploration. Previous work includes biologically inspired flock control as ants [12], formation flying control of airplanes [13], and unmanned airplane path planning [14]. In [15], multiple robots are interconnected via virtual spring and damper so that the movement of a robot influences the movement of other robots. In our model, the sensor nodes are interconnected with springs as in Figure 3.

**Figure 4.**
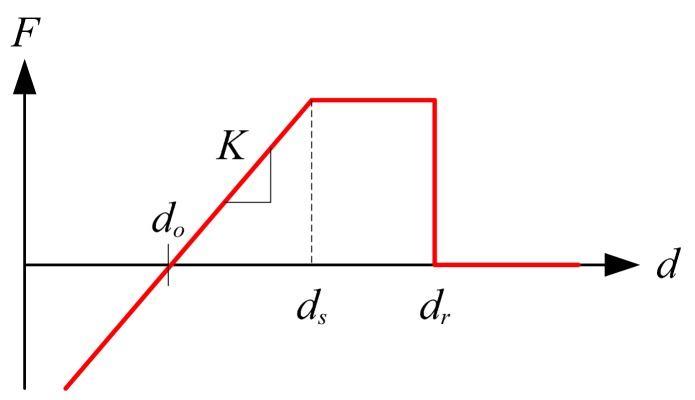
Spring constant characteristics.

The role of the springs is to set the appropriate distance between nodes because the locations of the nodes are at their spring force equilibrium state. Configuration of the nodes is based on the on-line tuning of the spring constants. The conventional spring generates the spring force proportional to the displacement. However, the spring used in this work has characteristics as shown in Figure 4.

The characteristics are expressed as:

(40) $$F=K(d-d_{o})\;\text{if}\;d<d_{s}$$

(41) $$F=F_{s}\;\text{if}\;d_{s}<d<d_{r}$$

(42) $$F=0\;\text{if}\;d_{r}<d$$

The initial spring length is *d_o_*. Therefore, the spring force is zero when the distance between two nodes is *d_o_*. If the other node is closer than *d_o_*, they push each other, while they pull each other if it is farther than *d_o_* up to *d_r_*. The spring force is set saturated for ranges of *d_s_* ∼ *d_r_*. No interaction is set farther than *d_r_*. The spring constant *K* and the offset value, *d_o_* are set appropriately so that the distance between nodes are controlled and thereby the density of nodes are as desired. The location of the node is updated as:

(43) $$X_{i,\text{new}}=X_{i,old}+\alpha_{i}\sum_{i\neq j}F_{i,j}$$

where *α_i_* is positive constant and *F_i_*_,_ *_j_* is the force from the spring interconnected between the *i*-th and the *j*-th node.

For verification of the proposed algorithm, a sensor network composed of 11 × 11 nodes is used. Initially, the node is evenly spaced and all the interconnected springs have the same value of 100 N/m. For simplicity, 40 nodes on the boundary line are set fixed. All nodes inside the boundary have right/left horizontal springs and top/bottom vertical springs inter-connected with neighbor nodes. In case the horizontal spring constant matrix and vertical spring constant matrix are changed as follows:

$$\begin{array}{c} K_{h}=\left[\begin{array}{cccccccccc} 100 & 100 & 300 & 300 & 100 & 100 & 100 & 100 & 100 & 100\\ 100 & 300 & 500 & 500 & 300 & 100 & 100 & 100 & 100 & 100\\ 100 & 300 & 500 & 500 & 300 & 100 & 100 & 100 & 100 & 100\\ 100 & 300 & 500 & 500 & 300 & 100 & 100 & 100 & 100 & 100\\ 100 & 100 & 300 & 300 & 100 & 100 & 60 & 60 & 100 & 100\\ 100 & 100 & 100 & 100 & 100 & 60 & 20 & 20 & 60 & 100\\ 100 & 100 & 100 & 100 & 100 & 60 & 20 & 20 & 60 & 100\\ 100 & 100 & 100 & 100 & 100 & 60 & 20 & 20 & 60 & 100\\ 100 & 100 & 100 & 100 & 100 & 100 & 60 & 60 & 100 & 100 \end{array}\right]\\ K_{v}=\left[\begin{array}{ccccccccc} 100 & 100 & 100 & 100 & 100 & 100 & 100 & 100 & 100\\ 100 & 300 & 300 & 300 & 100 & 100 & 100 & 100 & 100\\ 300 & 500 & 500 & 500 & 300 & 100 & 100 & 100 & 100\\ 300 & 500 & 500 & 500 & 300 & 100 & 100 & 100 & 100\\ 100 & 300 & 300 & 300 & 100 & 100 & 100 & 100 & 100\\ 100 & 100 & 100 & 100 & 100 & 60 & 60 & 60 & 100\\ 100 & 100 & 100 & 100 & 60 & 20 & 20 & 20 & 60\\ 100 & 100 & 100 & 100 & 60 & 20 & 20 & 20 & 60\\ 100 & 100 & 100 & 100 & 100 & 60 & 60 & 60 & 100\\ 100 & 100 & 100 & 100 & 100 & 100 & 100 & 100 & 100 \end{array}\right] \end{array}$$

**Figure 5.**
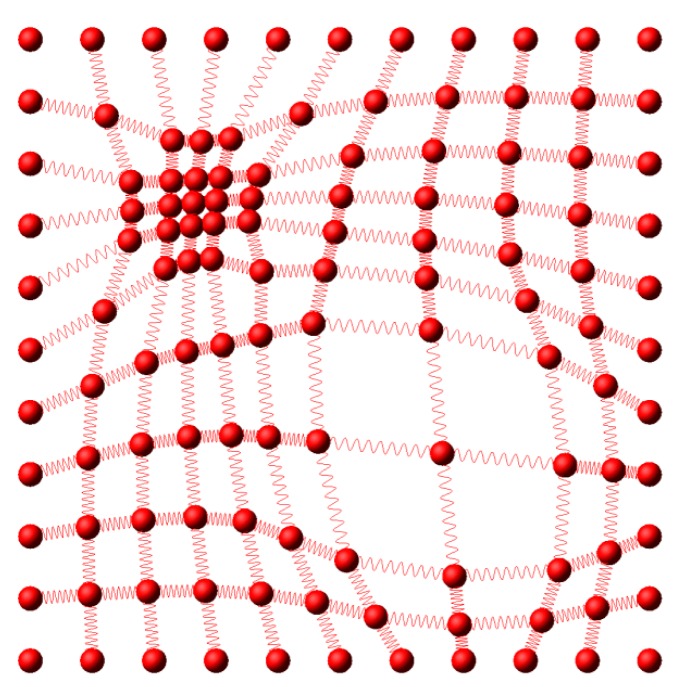
Configured sensor nodes.

The resulting configuration of the nodes is presented in Figure 5.

**Figure 6.**
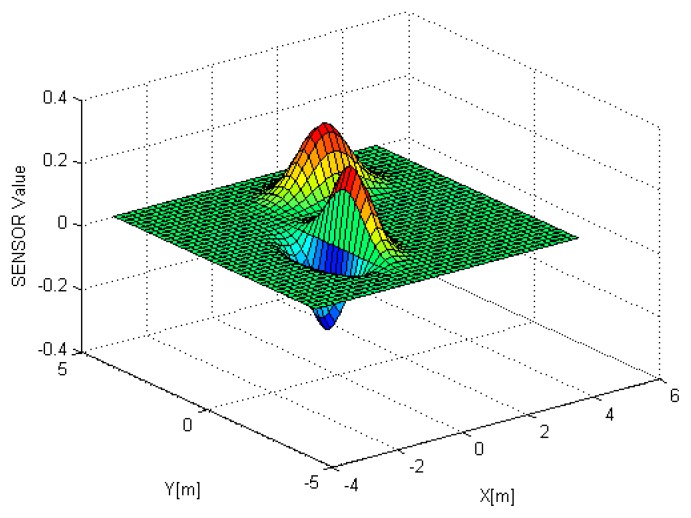
Environment model: surface plot.

For simulation, an environment is modeled as:

(44) $$z=xe^{\left(-3x^{2}-y^{2}\right)}+0.2\left(y-2\right)e^{\left(-{\left(x-2\right)}^{2}-2{\left(y-3\right)}^{2}\right)}$$

where *x* and *y* represent the location and *z* does the physical property to be measured, for instance, temperature. The model is shown in Figure 6.

**Figure 7.**
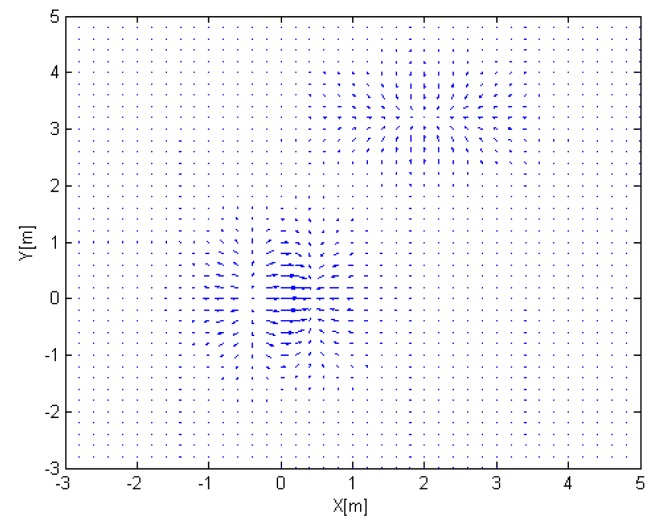
Spatial gradient.

The spatial gradient vectors, $\frac{\partial z}{\partial x}î+\frac{\partial z}{\partial y}ĵ$ are shown in Figure 7. The gradient is to be estimated by active sensing.

**Figure 8.**
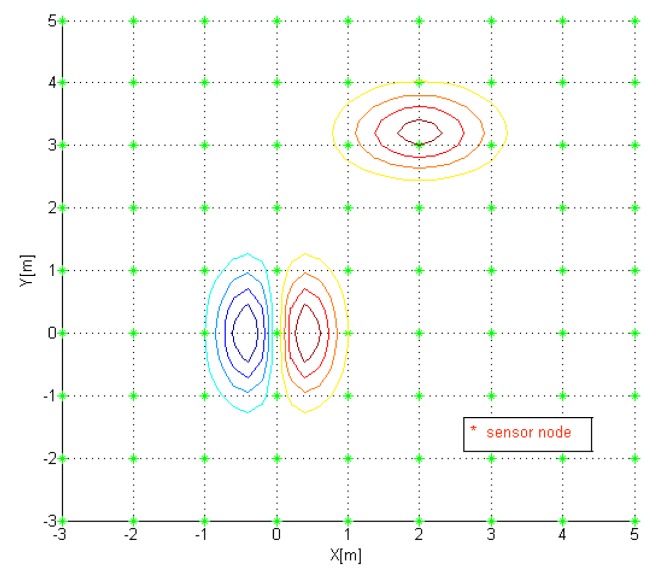
Initial Sensor Node Location.

A sensor network composed of 9 × 9 nodes is used, of which each node is evenly distributed. Each node is marked as *, in Figure 8.

**Figure 9.**
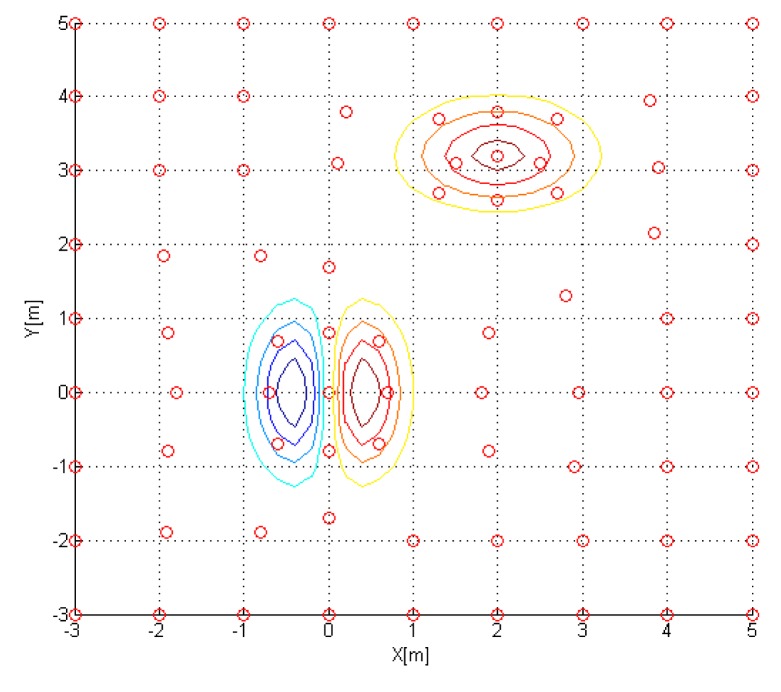
Updated sensor node location.

With the estimated gradients, each sensor node location is updated from Equations (40)–(43). The updated sensor node location is shown (marked as circle) in Figure 9. Note that 30 boundary nodes are set fixed.

**Figure 10.**
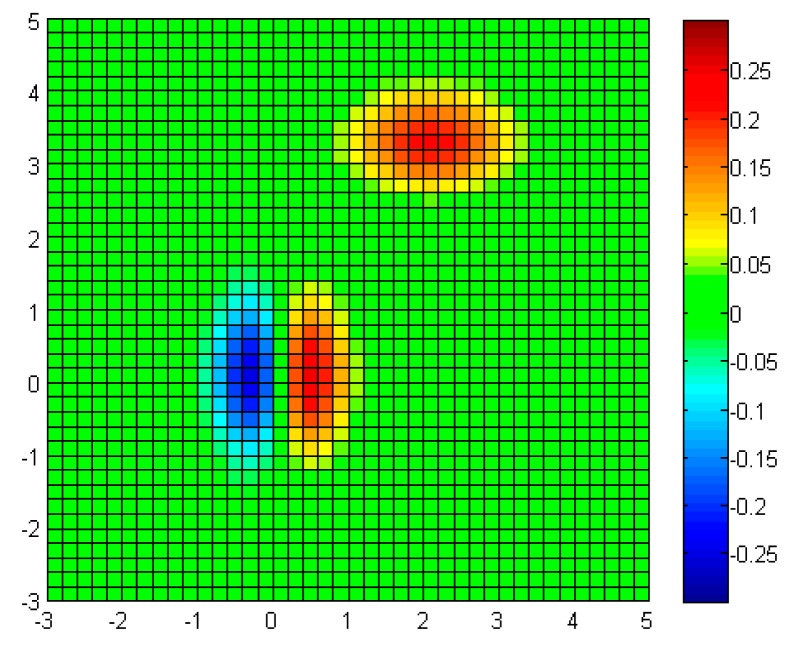
Environment model: 2D plot with color map.

**Figure 11.**
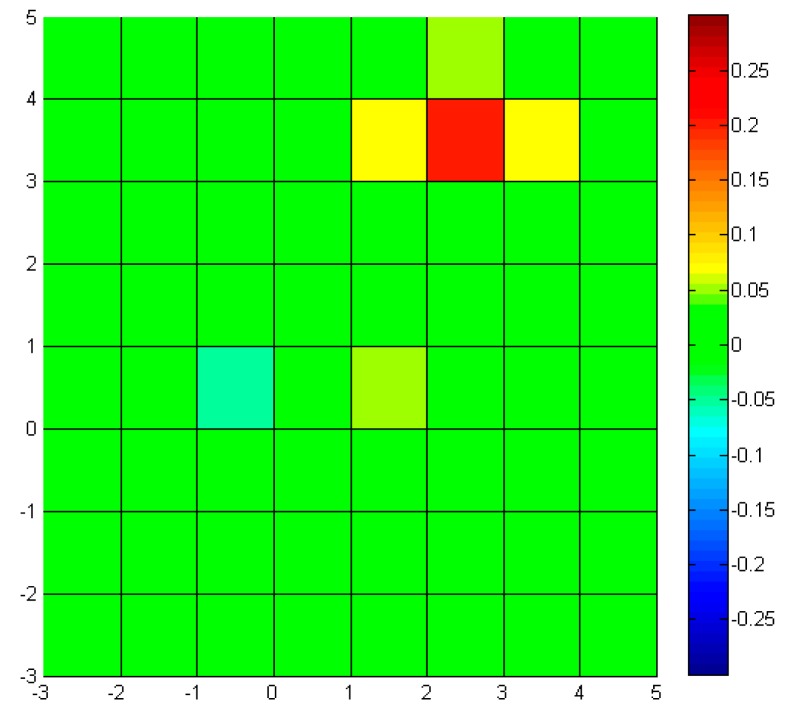
Measured model (initial): 2D plot with color map.

In order to show the effectiveness of the sensor node reconfiguration, the measured properties are compared in the following figures. For reference, the environment model is presented in 2D plot with color bar in Figure 10. The environment model is composed of 41 × 41 cells. The green cell shows the region with value of 0, while the red and blue shows higher and lower values respectively. With the initial (evenly distributed) sensor nodes, the measured properties are shown in Figure 11.

**Figure 12.**
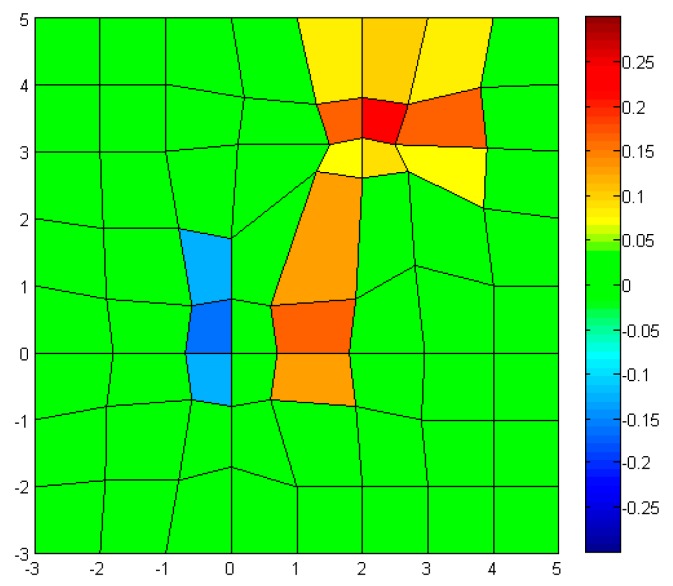
Measured model (reconfigured): 2D plot with color map.

Around (0,0), the measured values are quite different from the ones in the original model, due to small number of sensor nodes (9 × 9), *i.e.*, low resolution sampling. After the sensor nodes are reconfigured, the measured values are shown in Figure 12.

The measurements with the reconfigured sensor nodes show better representation than the initial one, even though the same number of sensor nodes are used. The iteration of the reconfiguration process will increase the sensing capability.

## 5. Conclusions/Outlook

A sensor network performs a very important role in environment monitoring especially in dangerous or isolated areas. For maximum sensing performance, it is necessary to place sensor nodes appropriately with a limited number of sensor nodes. The configuration of sensor nodes for an environment is presented. The location of each node is calculated from the spring connected node model. The spring constant is updated from the spatial gradient of the physical property. The estimation of the gradient is based on the active sensing. Instead of passively measuring the instant transducer outputs, planned inputs are applied to the system and the responses of the system are correlated with the given inputs. The correlation value was proven, that it is proportional to the gradient of the output with respect to the input. The simulation result is in accord with the idea that more nodes are to be placed in the region where the property changes are large while less nodes are needed, where the property changes are small or zero.
